# Effects of physiological self-crowding of DNA on shape and biological properties of DNA molecules with various levels of supercoiling

**DOI:** 10.1093/nar/gkv055

**Published:** 2015-02-04

**Authors:** Fabrizio Benedetti, Aleksandre Japaridze, Julien Dorier, Dusan Racko, Robert Kwapich, Yannis Burnier, Giovanni Dietler, Andrzej Stasiak

**Affiliations:** 1 Center for Integrative Genomics, University of Lausanne, 1015-Lausanne, Switzerland; 2 SIB Swiss Institute of Bioinformatics, 1015-Lausanne, Switzerland; 3 Institute of Physics of Biological Systems, École Polytechnique Fédérale de Lausanne (EPFL), 1015-Lausanne, Switzerland; 4 Vital-IT, SIB Swiss Institute of Bioinformatics, 1015-Lausanne, Switzerland; 5 Polymer Institute of the Slovak Academy of Sciences, 845 41 Bratislava, Slovakia; 6 Department of Medical Physics, University of Silesia in Katowice, 40-007 Katowice, Poland; 7 Institute of Theoretical Physics, École Polytechnique Fédérale de Lausanne (EPFL), 1015-Lausanne, Switzerland

## Abstract

DNA in bacterial chromosomes and bacterial plasmids is supercoiled. DNA supercoiling is essential for DNA replication and gene regulation. However, the density of supercoiling *in vivo* is circa twice smaller than in deproteinized DNA molecules isolated from bacteria. What are then the specific advantages of reduced supercoiling density that is maintained *in vivo*? Using Brownian dynamics simulations and atomic force microscopy we show here that thanks to physiological DNA–DNA crowding DNA molecules with reduced supercoiling density are still sufficiently supercoiled to stimulate interaction between cis-regulatory elements. On the other hand, weak supercoiling permits DNA molecules to modulate their overall shape in response to physiological changes in DNA crowding. This plasticity of DNA shapes may have regulatory role and be important for the postreplicative spontaneous segregation of bacterial chromosomes.

## INTRODUCTION

Numerous theoretical, computer simulation and experimental studies have shown that as the concentration of long circular polymers increases their equilibrium shapes change from swollen, spread out configurations to compressed globules (1–9). This contrasts with the properties of highly crowded linear polymers, which at equilibrium, can freely spread in the available volume (8). Concentration-induced compression of circular molecules is caused by topological effects that limit the penetrance of circular molecules into the space ‘encircled’ by other circular molecules (1–10).

Studies of circular polymers at high concentration focused mainly on the melt state, which is achieved when polymers’ concentration reach at least 50% of volume occupation (6,8). The melt state has high practical and commercial interests as many synthetic materials are made of polymers at melt state (11). However, the topological effects, although less pronounced, are also expected to play an important role at biologically significant polymer concentrations of 10–20% of volume occupation, as it is the case of the DNA in bacterial nucleoids (12) or of chromatin in cell nuclei (13). Bacterial chromosomes and bacterial plasmids are circular and as such should be a subject to concentration-induced compression resulting from topological effects. However, in addition to being circular, bacterial DNA is also supercoiled (14) and this may affect the process of concentration-induced compression. Numerous studies of supercoiled DNA molecules characterized their structure and properties at highly diluted conditions such as these used for electron microscopy, for example (15–18). However, this well-characterized structure may be significantly different from the biologically relevant structure of supercoiled DNA at the physiological self-crowding conditions.

Here we apply Brownian dynamics simulations and atomic force microscopy to investigate how non-supercoiled and supercoiled DNA molecules change their shape and other biologically relevant properties as the concentration of DNA increases up to the physiological DNA concentrations in bacterial cells. We are interested in three different situations: molecules with the supercoiling density (*σ*) of ca −0.05, −0.025 and 0. The supercoiling density of −0.05 indicates that DNA molecules have 5% lower linking number than they would have in torsionally unconstrained form and thus roughly show the linking deficit of 1 per each fragment of 200 bp.

The *σ* ≈ −0.05 corresponds to the supercoiling density observed in deproteinized plasmids isolated from bacterial cells such as these used for standard biophysical and biochemical experimentation with supercoiled DNA molecules (19). The second case (*σ* ≈ −0.025) corresponds to the supercoiling density of DNA plasmids within bacterial cells where due to binding of histone-like proteins to DNA its structure is changed in such a way that the supercoiling density decreases to a roughly 50% of the supercoiling density this DNA would have in the deproteinized form (19–22). We were interested to investigate what are possible advantages of this reduced supercoiling density *in vivo*. These advantages could explain why this specific supercoiling density is maintained in bacterial cells by very complex homeostatic regulatory mechanisms involving several types of DNA topoisomerases (23).

The third case (*σ* ≈ 0) corresponds to non-supercoiled molecules, which are used as a reference point in our studies and also allow us to make a comparison with other studies investigating the effect of high DNA concentration on relatively small circular polymer molecules (24–26). We simulate behaviour of DNA molecules of the size of 3 kb, which is the size of popular plasmids used for characterization of structure of supercoiled DNA molecules by spectroscopy methods (24,25,27) or by electron microscopy (17,18).

## MATERIALS AND METHODS

### Molecular dynamics simulations

The MD simulations were performed using HOOMD-blue software package [http://codeblue.umich.edu/hoomd-blue] (28) run on GPUs. Circular DNA molecules with the size of ca 3000 bp were modelled as semi-flexible beaded chains with flexular and torsional resistance. The diameter of each bead constituted one Lennard-Jones length unit (*σ*_LJ_) and corresponded to 3 nm, which approximates effective diameter of DNA under physiological conditions where electrostatic charges are nearly completely screened (29). All simulations were performed under periodic boundary conditions (PBC) and involved 20 independent circular molecules with 334 beads each. To reach the desired occupancy the PBC box was slowly reduced to the appropriate dimension.

A cut Lennard-Jones potential (with the repulsive part only) with *r*_cut_ = 1.0 *σ*_L-J_ was responsible for excluded volume interactions between individual beads. Our model included also harmonic bonding potential as well as bending and torsional potential. The parameters of the model were chosen so that the energy *ϵ*_0_ unit corresponded to 1 *k*_B_*T* (4 × 10^−21^ J). The bending stiffness was set to *ε*_b_ = 17.0 *ε*_0_, which results in the persistence length corresponding to 50 nm.

To implement the torsional stiffness we followed the approach presented in (30) except that we use two dihedral angles instead of one to better approximate the twist angle (see Supplementary Figure SI1–3 for more information). This approach is similar to one described recently by Brackley *et* *al*. (31). However, thanks to placing the frames of references for measuring dihedral angles at middle points between the centres of consecutive beads, our method has the advantage that it does not fail for 90° bending angles.

Studied supercoiling of Δ*L*_k_ = −7 and Δ*L*_k_ = −14 corresponded to the supercoiling density *σ* ≈ −0.025 and *σ* ≈ −0.05, respectively.

The analysed configurations (6 × 10^5^ to 13 × 10^6^, depending on the equilibration time of a given system) were taken every 1000 simulation steps over simulation runs that exceeded at least eight-fold the equilibration time of modelled system. The equilibration time was evaluated by monitoring the evolution of radii of gyration of individual molecules.

The interaction between enhancers and promoters was modelled as truncated Lennard-Jones potential surrounding enhancer beads with the well depth ϵ of 8 k_B_T and with r_cut set to two bead diameters (beyond the r_cut the potential is zero). The enhancer and promoter sites were placed 167 beads aside. Beads representing enhancers and promoters were considered in a contact when their surface-to-surface distance was smaller than bead's diameter.

### Preparation of non-supercoiled DNA

A 2.7 kb (pUC19 2686 bp) supercoiled plasmid DNA was purchased from Fermentas (Switzerland) and nicked using Nt.BstNBI nicking enzyme (NEB). Nicked plasmids were further extracted from 1.5% agarose gel and purified using extraction kit from Promega. DNA was then placed in TE buffer composed of 10 mM Tris and 1 mM ethylenediaminetetraacetic acid solution.

### AFM sample preparation and AFM imaging

All Atomic Force Microscopy (AFM) samples were prepared in the AFM Buffer consisting of 1 mM Tris and 4 mM MgCl_2_ (pH = 7.0). Concentrated aliquots of nicked DNA were mixed with AFM buffer to achieve final concentrations of 0.5 ng/μl and 2.2 ng/μl in 20 μl buffer volume. Twenty microlitres drops were then deposited on freshly cleaved mica for 5 min. Afterwards the mica was rinsed with 1 ml of double distilled water and dried under a gentle nitrogen flow.

AFM images were collected using a MultiMode Scanning Probe Microscope (SPM) with a Nanoscope III controller (Veeco Instruments, Santa Barbara, CA, USA) operated in tapping-mode in air. The AFM cantilevers used in air had a spring constant of 5 N/m (Veeco cantilevers, TAP150A) with resonance frequencies ranging between 120 and 160 kHz. All recorded AFM images consisted of 512 × 512 pixels with scan frequency ≤1 Hz. Images were simply flattened using the Gwyddion software (32) n (Version 2.25) and no further image processing was carried out.

### DNA tracing

AFM images were analysed using ‘DNA Trace’, which is a home-made Java based analysis software (33). Once the DNA molecules were traced the software was also used to calculated their length and asphericity. Analysis of DNA molecules at high density of deposition was not considering the cases where one or more molecules were nested in another circular DNA molecule (34). In these specific cases one is not dealing with the topological exclusion but rather with the topological inclusion.

## RESULTS

### Crowding-induced changes in overall shape of circular, non-supercoiled and supercoiled DNA molecules

**Figure 1.**
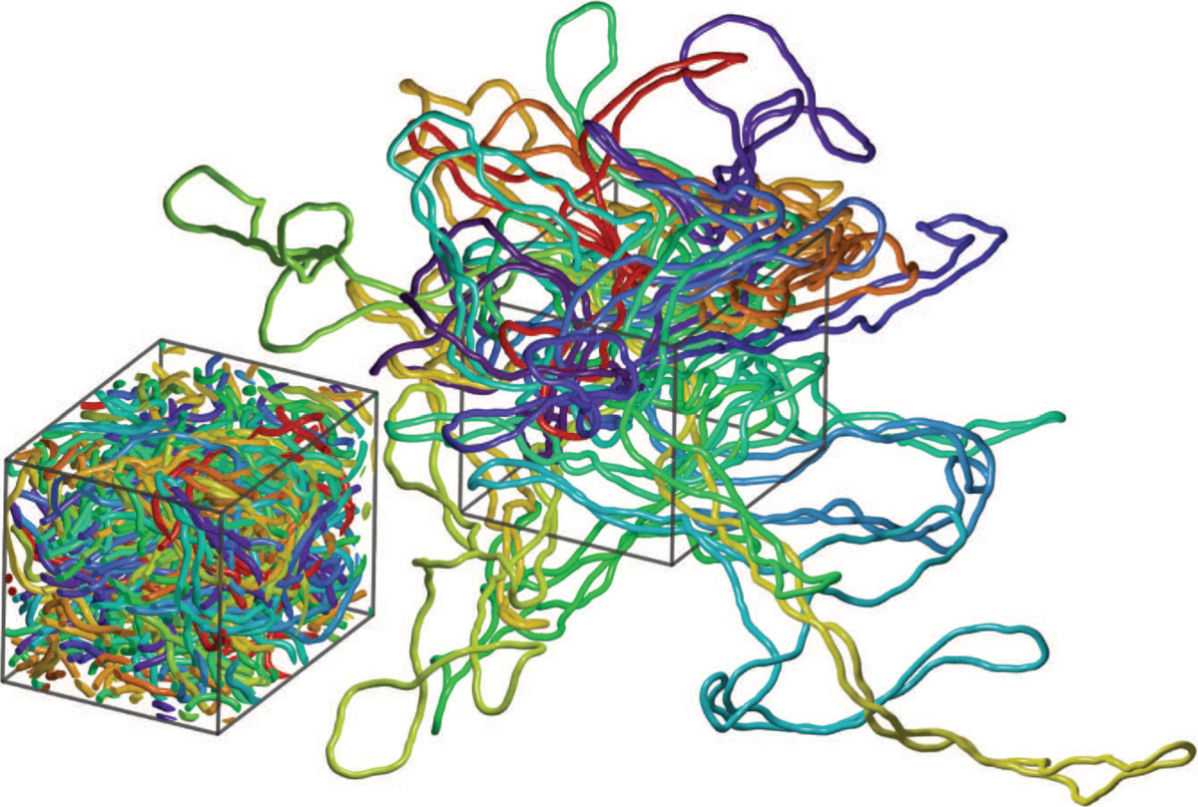
Snapshots from molecular dynamics simulations, which use periodic boundary conditions (PBC) to study the effect of 20% crowding on non-supercoiled DNA molecules. The periodic box image (shown as an inset) allows to appreciate how crowded are DNA molecules at 20% volume occupation. The main image shows 20 individual simulated DNA molecules that extend from one periodic box over several neighbouring ones. These molecules are shown upon making all their periodic copies invisible.

As mentioned above, the concentration of DNA in bacterial nucleoids reaches 20% of volume occupation (12). Figure 1 presents a snapshot from our simulation of 20 non-supercoiled plasmids (each 3 Kb long) at 20% volume occupation. The inset shows a snapshot of the periodic box of the simulation together with all the fragments of 20 simulated molecules that got ‘permutated’ within one periodic box. Such an image of the periodic box helps us to appreciate how crowded are the DNA molecules at 20% volume occupation. However, an image of one periodic box is unsuited to visualize shape of individual simulated DNA molecules that extend over several periodic boxes. To visualize and analyse shapes taken by individual molecules it is necessary to trace individual molecules over several periodic boxes. The main image presents such ‘tracings’ or reconstructions of the 20 simulated independent molecules extending from one periodic box. This image presents very well the shape of individual molecules but may give an impression that the molecules extend from the crowded box into unoccupied space. This is however not the case as due to periodic nature of the simulation procedure the entire space is as crowded as shown in the inset. This crowding is not visible in the main image since all periodic copies are eliminated. The individual reconstructions of simulated molecules such as these shown in Figure 1 are then analysed by us to study crowding-induced changes of shapes of simulated, circular DNA molecules.

Shapes of complex objects such as of momentary configurations of polymers are conveniently described by considering characteristic ellipsoids of inertia defined by the distribution of mass in individual analysed configurations (35–38). The considered ellipsoids have the same mass and the same rotational moments of inertia as the momentary configurations of the modelled polymers that they represent, assuming that these momentary configurations are absolutely rigid and that the mass of every bead in the modelled chains is the same. The dimensions of characteristic inertial ellipsoids are computed assuming that all their masses are equally spread on their surface (39).

**Figure 2.**
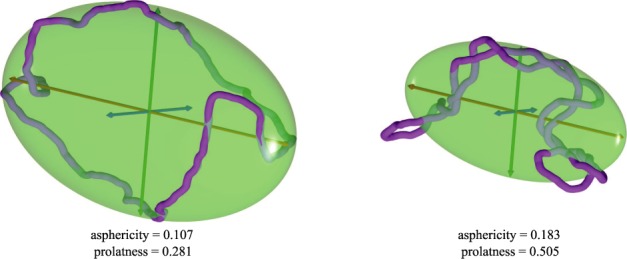
Examples of simulated momentary configurations of non-supercoiled (left) and supercoiled DNA molecules (right) together with their characteristic ellipsoids of inertia. The three principal axes of rotation a, b and c, where a ≥ b ≥ c, are shown as red, green and blue, respectively. The values of their asphericity and prolateness are indicated. The shown configurations have typical shapes obtained in simulations of diluted non-supercoiled and supercoiled (*σ* ≈ −0.05) DNA molecules, respectively. Their aspericity and prolatness values are both close to the corresponding average values for this type of simulated DNA molecules (see Figures 3a and c, and 4 a and c). Notice that the supercoiled DNA molecule is more aspherical and more prolate than the non-supercoiled molecule.

Figure 2 shows examples of typical configurations of non-supercoiled (left) and supercoiled (right) DNA molecules (*σ* ≈ −0.05) obtained in simulations of non-crowded molecules. The configurations are shown together with their characteristic inertial ellipsoids. It is visible that supercoiling makes the molecules and their characteristic inertial ellipsoids more compact. Supercoiled molecules also have more elongated shape when one considers aspect ratios of the ellipsoids. To provide more quantitative measures of the overall shape of the analysed configurations the ratios between the three semiaxes length (a, b and c where a ≥ b ≥ c) of the characteristic inertial ellipsoids are used to calculate such shape characteristics as asphericity and prolatness (39).

The asphericity measures how much the shape of a given object differs from a sphere. Perfect sphere has the asphericity value of 0, whereas a straight thin rod has the asphericity value very close to 1.

The asphericity values were calculated according to the formula:

\begin{equation*} A(a,b,c) = \frac{{(a - b)^2 + (a - c)^2 + (b - c)^2 }}{{2(a + b + c)^2 }} \end{equation*}

where a, b and c are the lengths of principal semiaxes of characteristic ellipsoids of inertia (39).

**Figure 3.**
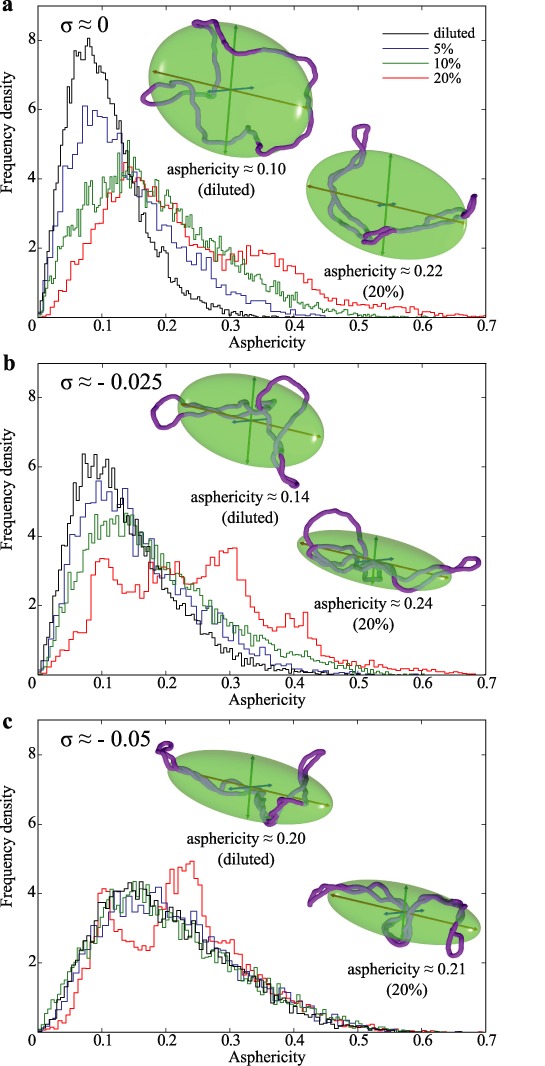
Physiological self-crowding strongly increases the asphericity of non-supercoiled DNA but affects less the asphericity of supercoiled DNA molecules. Individual density profiles of asphericity values were obtained after analysis of more than 12 000 momentary configurations representing three types of simulated DNA molecules: non-supercoiled (**a**), supercoiled with *σ* ≈ −0.025 (**b**) and *σ* ≈ −0.05 (**c**) at four different DNA concentrations: diluted, 5, 10 and 20%. Insets show simulated configurations at diluted state (left) and at physiological DNA concentration of 20% (right). The shown configurations have their asphericity values very close to the average values observed in simulations of the corresponding system.

Figure 3 compares the crowding-induced changes in asphericity in non-supercoiled (panel a) and supercoiled plasmids with *σ* ≈ −0.025 or *σ* ≈ −0.05 (panels b and c, respectively). Each of the shown frequency density profiles is obtained after analysis of at least 12 000 momentary configurations that were saved after every 1000 steps of simulations, where each simulation run involved 20 independent DNA molecules. Insets in each panel show configurations (together with their ellipsoids of inertia) typical for a given type of DNA molecules when these were diluted (left) or subject to crowding resulting from physiological DNA concentration of 20% (right). As typical, we considered here configurations that show asphericity values that are very close to the average asphericity values for a given type of simulated DNA molecules at given conditions.

Panel a shows that non-supercoiled circular DNA molecules become significantly more aspherical with increasing DNA concentration. The average asphericity values for diluted non-supercoiled DNA circles is ca 0.10 but it changes to ca 0.22 as DNA concentration reaches 20%.

Comparison between frequency density profiles and typical configurations shown in panels a, b and c reveals that diluted molecules strongly increase their asphericity as a result of supercoiling. However, supercoiled molecules are more resistant to further increase of asphericity due to growing DNA concentration. This effect is especially evident for molecules with *σ* ≈ −0.05.

The asphericity values tell us how strongly the configurations differ from a perfect spherical symmetry but they do not reveal whether the configurations are better approximated by prolate (such as rugby ball) or oblate ellipsoids (such as M&M candy). To obtain this information one needs to calculate values of prolatness according to the formula:

\begin{equation*} P(a,b,c) = \frac{{(2a - b - c)(2b - a - c)(2c - a - b)}}{{2(a^2 + b^2 + c^2 - ab - ac - bc)^{3/2} }} \end{equation*}

Prolatness values can range from −1 for perfectly oblate ellipsoids (a = b > c), to 1 for perfectly prolate ellipsoids (a > b = c). The negative prolatness values indicate that the configurations and their ellipsoids of inertia are oblate ((a-b) < (b-c)) whereas positive values characterize prolate shapes ((a-b ) > (b-c)).

**Figure 4.**
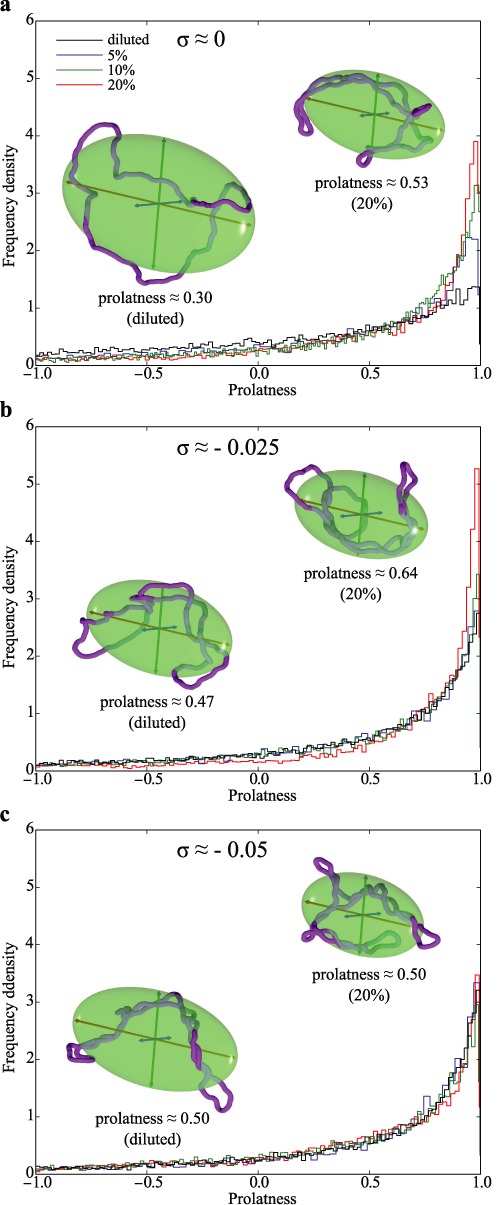
Physiological self-crowding increases prolatness of non-supercoiled DNA whereas the prolatness of supercoiled DNA molecules with *σ* ≈ −0.05 remains practically unchanged. Frequency density profiles of prolatness values were calculated for the same statistical samples as these analysed in Figure 3. Data for non-supercoiled DNA molecules and supercoiled with *σ* ≈ −0.025 and *σ* ≈ −0.05 are presented in **a**, **b** and **c**, respectively. Insets show typical configurations of corresponding types of molecules when these were diluted (left) or subject to crowding resulting from 20% DNA concentration (right). The shown configurations have their prolatness values very close to the average values observed in simulations of the corresponding system.

Figure 4 compares the concentration-induced changes of prolatness in non-supercoiled and supercoiled plasmids with *σ* = −0.025 or *σ* = −0.05 (a, b and c, respectively). Insets in each panel show configurations with the average prolatness for diluted (left) and 20% concentrated DNA molecules (right). It is well visible that non-supercoiled molecules (Figure 4a), which have a significant proportion of oblate shapes at low DNA concentration, strongly increase their overall prolatness and show an increasing number of molecules with high prolatness (a > b ≈ c) upon rising DNA concentration. Supercoiled DNA molecules with *σ* ≈ −0.025 (Figure 4b), which already at diluted conditions have the average prolatness value similar to this of non-supercoiled DNA at elevated DNA concentration, show a substantial further increase of prolatness as DNA concentration rises to 20%. The average prolatness of supercoiled DNA molecules with *σ* ≈ −0.05 (Figure 4c) is practically unchanged by an increase of DNA concentration and is very similar to the average prolatness of non-supercoiled DNA molecules at high crowding.

### Concentration-induced changes of the local intra-molecular thickness in non-supercoiled and supercoiled DNA molecules

Data presented in Figures 3 and 4 revealed that non-supercoiled DNA molecules showed large changes of shape upon increasing DNA concentration. However, in supercoiled DNA the extent of these shape changes decreased with increasing magnitude of supercoiling density. To characterize more precisely the effect of DNA–DNA crowding, we focused on local intra-molecular thickness of simulated molecules.

To determine the local intra-molecular thickness in analysed configurations we were inspired by the ref. (40). For analysed configurations we calculated first local tangent directions. Subsequently, we asked what is the largest possible sphere that is tangential to a given local direction and which can be put at any azimuthal angle around the local direction without intersecting with the rest of the chain. To eliminate the effect of regions with high local curvature, we have neglected short portions of the chain surrounding the point of tangency when checking for the intersection between the probing sphere and the rest of the chain.

**Figure 5.**
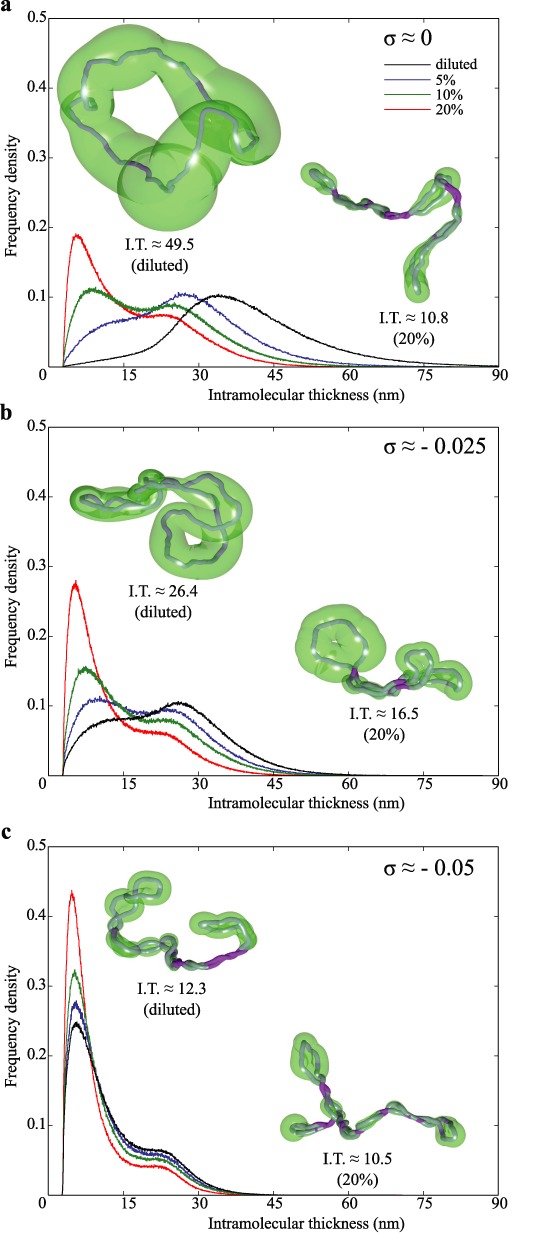
Physiological crowding of DNA molecules decreases their local intra-molecular thickness. Frequency density profiles of intra-molecular thickness are calculated for individual beads within the same large sets of molecules as these analysed in Figures 3 and 4. Insets show typical configurations for diluted molecules (left) and molecules subject to 20% crowding (right). The intra-molecular thickness is visualized in the form of semi-transparent tubes with varying diameter. The tube diameter at a given region corresponds to the intra-molecular thickness at that region. Notice that in regions where portions of molecules brought into a close apposition touch each other the intra-molecular thickness corresponds to the actual diameter of modelled DNA.

Figure 5a–c presents frequency density profiles of local intra-molecular thickness calculated for individual beads in all analysed configurations representing a given type of molecules and a given DNA concentration (see figure legends). Insets show typical configurations of corresponding simulated DNA molecules together with surrounding tubes whose varying diameter corresponds to the local thickness a given molecule shows at a given region. The shown configurations are typical for diluted (left) and 20% concentrated (right) conditions i.e. their average intra-molecular thickness are very close to the average intra-molecular thickness for the corresponding class and conditions of simulated DNA molecules.

The frequency density profiles in Figure 5a show how the local intra-molecular thickness changes in non-supercoiled molecules as their concentration increases. When molecules are diluted the local thickness is generally large and long portions of individual chains have the intra-molecular thickness exceeding 15-fold their own diameter (see the black frequency density profile and the configuration shown on the left). The frequency density profile shows unimodal distribution that can be expected from fluctuating circular molecules that are not crowded. However, as the concentration of modelled molecules increases they reduce their local thickness. For crowding resulting from 10 and 20% DNA concentration the frequency density profiles show two peaks. The stronger peak for the intra-molecular thickness, which corresponds to about two diameters of modelled DNA molecules, results from long regions where the segments of elongated circular molecules are in close apposition (see the right inset). The second peak or shoulder corresponds to intra-molecular-thickness ranging from 6 to 9 bead diameters of modelled molecules. That peak arises due to the fact that bending resistance of modelled molecules creates apical loops in crowded DNA molecules and in these loops there is no close contacts with other segments of the same molecule (see the right inset).

Figure 5b shows how the local thickness changes in simulated supercoiled DNA molecules with *σ* ≈ −0.025. At high dilution there is a broad distribution of thickness with the peak values of about nine geometrical diameters of the modelled DNA molecules and a shoulder with intra-molecular thickness of about 2 diameters of the modelled DNA. That shoulder in the frequency density of intra-molecular thickness reflects the fact that these molecules are supercoiled and have regions of extended close appositions. However, due to their low density of negative supercoiling these molecules also have regions that are relatively open and thus have relatively large intra-molecular thickness (see the left inset). Figure 5b also shows that as the concentration of simulated supercoiled DNA molecules with *σ* ≈ −0.025 increases the molecules get reshaped. There is an increase of length of the portions with close intra-molecular appositions, whereas the open apical loops progressively shrink in size.

Figure 5c analyses concentration-induced changes of the local intra-molecular thickness in supercoiled DNA molecules with *σ* = −0.05. One can see that these relatively strongly supercoiled molecules do not need to be compressed by other molecules to reach the average intra-molecular thickness of about 4 diameters of modelled molecules. When the concentration of strongly supercoiled molecules increases their average intra-molecular thickness slightly decreases to about 3.5 diameters of modelled DNA but this is mainly achieved by decreasing the size of apical loops rather than by further tightening of superhelical regions.

### DNA–DNA crowding increases the magnitude of writhe in supercoiled DNA molecules

**Figure 6.**
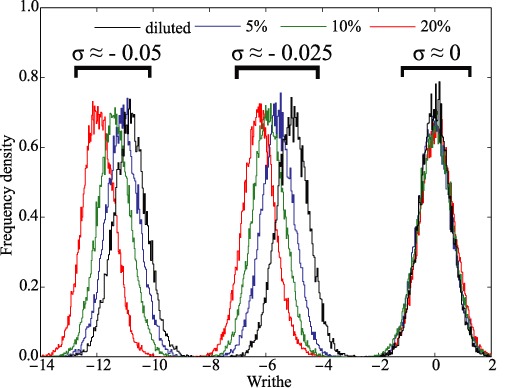
Physiological crowding of supercoiled DNA molecules increases the magnitude of their writhe. Probability density profiles of writhe were obtained after measuring writhe of individual molecules in the same statistical samples as these analysed in Figures 3–5.

Figure 5 showed that with increasing concentration of modelled DNA molecules their local intra-molecular thickness decreases. This effect is similar to the effect of increasing concentration of counterions that screen electrostatic repulsion between DNA segments (17,18). In that latter case it is of course not needed that DNA molecules are highly concentrated. Earlier studies showed that counterion-induced decrease of effective diameter in supercoiled DNA molecules results in more tight structure of plectonemic windings and an increase in the magnitude of writhe (17). Therefore, we have decided to investigate how the inter-molecular DNA–DNA crowding changes the writhe of supercoiled DNA molecules. Figure 6 illustrates that the magnitude of writhe of supercoiled DNA molecules increases with increasing DNA concentration and this applies to simulated molecules with both tested supercoiling densities. The increase of the magnitude of writhe makes it that the DNA decreases its torsional stress. This can protect supercoiled DNA *in vivo* from torsional tension-induced melting or transitions to alternative DNA structures (41). The decrease of torsional stress is also likely to prevent excessive formation of R-loops and thus decrease genome instability (42).

As could be expected, for modelled non-supercoiled DNA there was no change of writhe resulting from inter-molecular crowding. It is important to add here that neutron diffraction studies of supercoiled DNA molecules also indicted that the magnitude of writhe increases as the concentration of the molecules becomes sufficiently large to induce liquid crystal formation (25).

### Effect of DNA–DNA crowding on intra-molecular enhancer–promoter interactions

**Figure 7.**
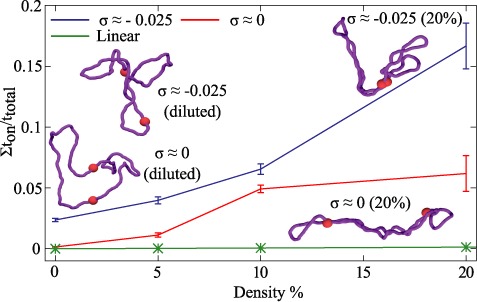
Physiological crowding of DNA molecules stimulates interaction between cis-regulatory elements in circular DNA molecules. The fraction of time during which enhancer and promoter sites with the mutual affinity of 8 k_B_T interact together grows with increasing DNA concentration both for non-supercoiled and supercoiled DNA molecules (*σ* ≈ −0.025). At all tested DNA concentrations the physiological supercoiling density (*σ* ≈ −0.025) was additionally increasing the fraction of molecules in which enhancers and promoters were in a contact. The insets show snapshots of non-supercoiled (two lower configurations) and supercoiled DNA molecules (*σ* ≈ −0.025) (two upper configurations), which were simulated under conditions corresponding to diluted state or 20% concentration (configuration shown on the left- and right-hand side of the figure, respectively). Configurations where enhancers interacted with promoters were most frequently observed in simulations of supercoiled DNA molecules (*σ* ≈ −0.025) at 20% concentration (see the configuration shown in the upper-right corner). Enhancer–promoter interactions in linear DNA molecules were practically not affected by DNA crowding (Linear) despite the fact that the affinity and genomic separation between enhancer and promoter sites were the same as in the tested circular non-supercoiled and supercoiled DNA molecules.

One of the important functions of DNA supercoiling is stimulation of interactions between DNA sites located within the same supercoiled DNA molecules (43–45) (46,47). Using Brownian dynamics simulations we have studied earlier the effect of progressive increase of supercoiling density on the interaction between enhancer and promoter sites with mutual affinity to each other (48). We have observed that the fraction of time during which modelled enhancer and promoter sites interacted with each other was growing with increasing supercoiling density (48). These earlier studies prompted us to investigate the effect of increasing self-crowding of DNA molecules on the interaction between enhancer and promoter sites. We were especially interested in the comparison between the situation where enhancer and promoter sites are in the same DNA molecule that either has supercoiling density typical for *in vivo* situation in bacterial cells (*σ* ≈ −0.025) or are non-supercoiled. Figure 7 allows us to compare how DNA–DNA self-crowding stimulates the interaction between sites with mutual affinity in non-supercoiled and moderately supercoiled DNA molecules. In both cases the tested enhancer–promoter affinity was set to 8 k_B_T, which is within the range tested by us in earlier simulation studies (48). We can see that self-crowding stimulates interaction between enhancer and promoter sites located in the same molecule and that this applies both to non-supercoiled and to supercoiled DNA molecules (*σ* ≈ −0.025). However, for each tested DNA concentration the fraction of time, during which enhancer and promoter interacted with each other was significantly larger in supercoiled DNA molecules. The stimulation of enhancer–promoter interaction by crowding is limited to circular DNA molecules that strongly change their shape due to topological exclusion. Linear DNA molecules with the same genomic distance and the same affinity between enhancer and promoter sites as in circular DNA molecules were practically unaffected by crowding (see the profile for linear DNA on Figure 7).

Observed here stimulation of enhancer promoter interaction by supercoiling is consistent with earlier experimental studies testing the effect of DNA supercoiling on enhancer–promoter interactions (46) and with earlier simulation studies (48). However, it was not established before that enhancer–promoter interactions are very sensitive to topological effects acting in concentrated circular DNA molecules but not in concentrated linear DNA molecules.

### Experimental observation of changes of DNA shape resulting from strong self-crowding

**Figure 8.**
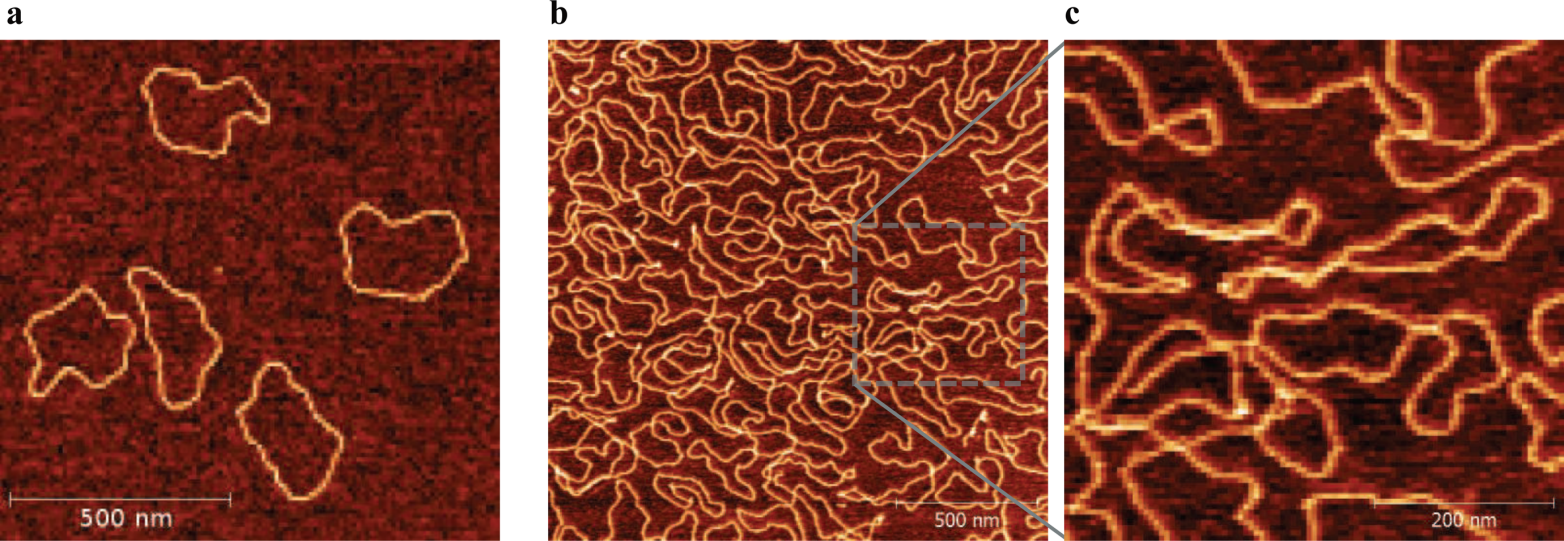
AFM visualization of 2D topological exclusion acting on non-supercoiled DNA molecules. Non-supercoiled DNA molecules were gently adsorbed to mica surface under conditions resulting in a low (**a**) or high (**b** and **c**) density of deposition. Notice that at high density of deposition most of the molecules are much more elongated than at the low density of deposition. Panel (c) shows at higher magnification a region demarked in panel (b).

We have used numerical simulations to study the effect of DNA crowding on circular supercoiled and non-supercoiled DNA molecules. Although we can be fairly confident that our simulations reflect what happens when the DNA concentration is increased, it is of course important to confirm simulations by direct observations. Direct observation of shapes of DNA molecules in highly concentrated bulk solutions is very difficult and would necessitate such techniques like cryo-fracture of highly concentrated DNA solutions, which could permit tracing of fragments of molecules (49). It is however relatively easy to observe and analyse shapes of entire DNA molecules experiencing 2D self-crowding as it is the case of DNA molecules adsorbed to the surface of mica used for AFM specimens. Since supercoiled DNA molecules hardly change their shape in response to increasing crowding, we decided to concentrate on nicked and thus non-supercoiled DNA molecules with the size (2.7 kb), which roughly corresponds to the size of DNA molecules simulated by us (3 kb). Figure 8 shows AFM images illustrating the effect of crowding on non-supercoiled DNA molecules. Comparing preparations with low and high density of DNA deposition (Figure 8a and b, respectively) it is clearly visible that 2-D crowding causes overall elongation of non-supercoiled DNA molecules in a way analogous to what was observed in our simulations of highly concentrated non-supercoiled DNA in bulk solutions (See insets in Figures 2a and 4a). Also calculation of asphericity of imaged molecules indicated that ellipses approximating their shapes become significantly more elongated with increasing DNA concentration (see Table 1). For 2D situation to calculate the asphericity we used the formula *A* = (a – b)^2^/(a + b)^2^ where a and b are the small and large principal axes of the radius-of-gyration tensor calculated for each trajectory (34,50). Although, the AFM studies investigated the effect of DNA self-crowding in 2D situation the obtained results provide experimental support of our simulations investigating 3D crowding of circular DNA molecules.

**Table 1.** AFM-determined 2D asphericity in diluted and concentrated non-supercoiled DNA molecules

Concentration	Number of analysed molecules	Length [nm]	Asphericity
0.5 ng/μl	95	800 ± 25	0.57 ± 0.2
2.2 ng/μl	60	820 ± 20	0.65 ± 0.2

## DISCUSSION

Using Brownian dynamics simulations and atomic force microsopy we have investigated how the shape of circular supercoiled and non-supercoiled DNA molecules is affected by topological exclusion caused by high concentration of circular DNA molecules. We have observed that as the DNA concentration increases, up to the physiological concentration of 10–20%, such as DNA concentrations within bacterial nucleoids, the molecules increase their asphericity and prolatness (see Figures 3 and 4). We have shown that self-crowding of supercoiled DNA molecules acts in a similar way as increasing effective density of supercoiling in non-crowded DNA molecules. In both cases the writhe of molecules increases and in both cases the intra-molecular interactions such as these occurring between enhancers and promoters are facilitated (48). Our results show that concentration-induced tightening of supercoiled DNA molecules with *σ* ≈ −0.025 partially compensates for the fact that the effective linking deficit of supercoiled DNA in bacterial cells amounts to only half of what is observed in deproteinized plasmids (19–22). Although we have studied relatively short DNA molecules the concentration-induced tightening of supercoiled DNA molecules is likely to apply to *in vivo* observed clusters of natural DNA plasmids (51) and to bacterial chromosomes that are both subject to similar crowding within bacterial cells. Large supercoiled DNA molecules naturally divide into branches, which effectively resemble smaller plasmids (52). *In vivo* studies have shown that bacterial chromosomes are supercoiled and are divided into relatively small branches (53), which are subject to a strong self-crowding (54). It is important to notice that supercoiled DNA molecules with *σ* ≈ −0.025 showed larger shape modulation by the topological exclusion resulting from their increasing concentrations than this was the case of non-supercoiled or supercoiled DNA molecules with the supercoiling density typical for deproteinized bacterial plasmids (*σ* ≈ −0.05). This ability of shape modulation by topological exclusion may play an important role in mechanical properties of bacterial nucleoids and be involved in reshaping of bacterial nucleoids during bacterial growth (55), in driving spontaneous segregation of replicated bacterial chromosomes (56) as well as in shape modulation resulting from double-strand breaks (57). Presumably, the intermediate level of supercoiling density in bacterial nucleoids *in vivo* (*σ* ≈ −0.025) was selected during the evolution as on one hand it is strong enough to stimulate interaction between cis regulatory elements and on the other hand it is weak enough to permit the DNA molecules to change their overall shape in response to physiological changes in DNA–DNA crowding (58).

It should be mentioned here that earlier numerical simulation studies showed that relatively short non-supercoiled circular polymer molecules react to their increasing concentrations by adopting elongated shapes (26,59). Such changes of shapes of small non-supercoiled circular DNA molecules were predicted theoretically (8) and also concluded from neutron scattering studies of concentrated DNA solutions (24,25). We are not aware though of earlier numerical simulation studies, investigating the effect of topological exclusion by following the equilibration of systems composed of many supercoiled DNA molecules interacting with each other. Earlier numerical approaches to study the effect of crowding on supercoiled DNA molecules were based on simulations of individual molecules subject to potentials mimicking crowding by other molecules such as nematic and depletion attraction potentials (60). However, setting the strength of these potentials is currently somewhat arbitrary as we still lack sufficient understanding of phenomena happening in solutions of supercoiled DNA at given elevated concentration and at given supercoiling density. Our current study is a step toward better understanding of these phenomena.

## SUPPLEMENTARY DATA

Supplementary Data are available at NAR Online.

SUPPLEMENTARY DATA
