# Supplementary material for: Effects of physiological self-crowding of DNA on shape and biological properties of DNA molecules with various levels of supercoiling
Source: Nucleic Acids Res. 2015 Feb 4;43(4):2390–9. doi: 10.1093/nar/gkv055 (PMC4344501; doi:10.1093/nar/gkv055)
Supplement: SUPPLEMENTARY DATA [file supp_43_4_2390__index.html]

Effects of physiological self-crowding of DNA on shape and biological properties of DNA molecules with various levels of supercoiling — Effects of physiological self-crowding of DNA on shape and biological properties of DNA molecules with various levels of supercoiling — SUPPLEMENTARY DATA 

# Effects of physiological self-crowding of DNA on shape and biological properties of DNA molecules with various levels of supercoiling

## SUPPLEMENTARY DATA

**Files in this Data Supplement:**

- SUPPLEMENTARY DATA
