# Supplementary material for: Effects of physiological self-crowding of DNA on shape and biological properties of DNA molecules with various levels of supercoiling
Source: Nucleic Acids Res. 2015 Feb 4;43(4):2390–9. doi: 10.1093/nar/gkv055 (PMC4344501; doi:10.1093/nar/gkv055)
Supplement: SUPPLEMENTARY DATA [file supp_gkv055_nar-03641-f-2014-File010.docx]

**Effects of physiological self-crowding of DNA on shape and biological properties of DNA molecules with various levels of supercoiling**.

Fabrizio Benedetti^a^*, Aleksandre Japaridze^b^*, Julien Dorier^a,c^, Dusan Racko^a,d^, Robert Kwapich^b,e^, Yannis Burnier^a,f^, Giovanni Dietler^b^ & Andrzej Stasiak^a^

^a^Center for Integrative Genomics, University of Lausanne, 1015-Lausanne, Switzerland, ^b^Institute of Physics of Biological Systems, École Polytechnique Fédérale de Lausanne (EPFL), 1015-Lausanne, Switzerland, ^c^ Vital-IT, SIB Swiss Institute of Bioinformatics, 1015-Lausanne, Switzerland, ^d^Polymer Institute of the Slovak Academy of Sciences, 842 36 Bratislava, Slovakia, ^e^Department of Medical Physics, University of Silesia in Katowice, 40-007 Katowice, Poland, ^f^Institute of Theoretical Physics, École Polytechnique Fédérale de Lausanne (EPFL), 1015-Lausanne, Switzerland.

* Joint first authors

Supplementary Data


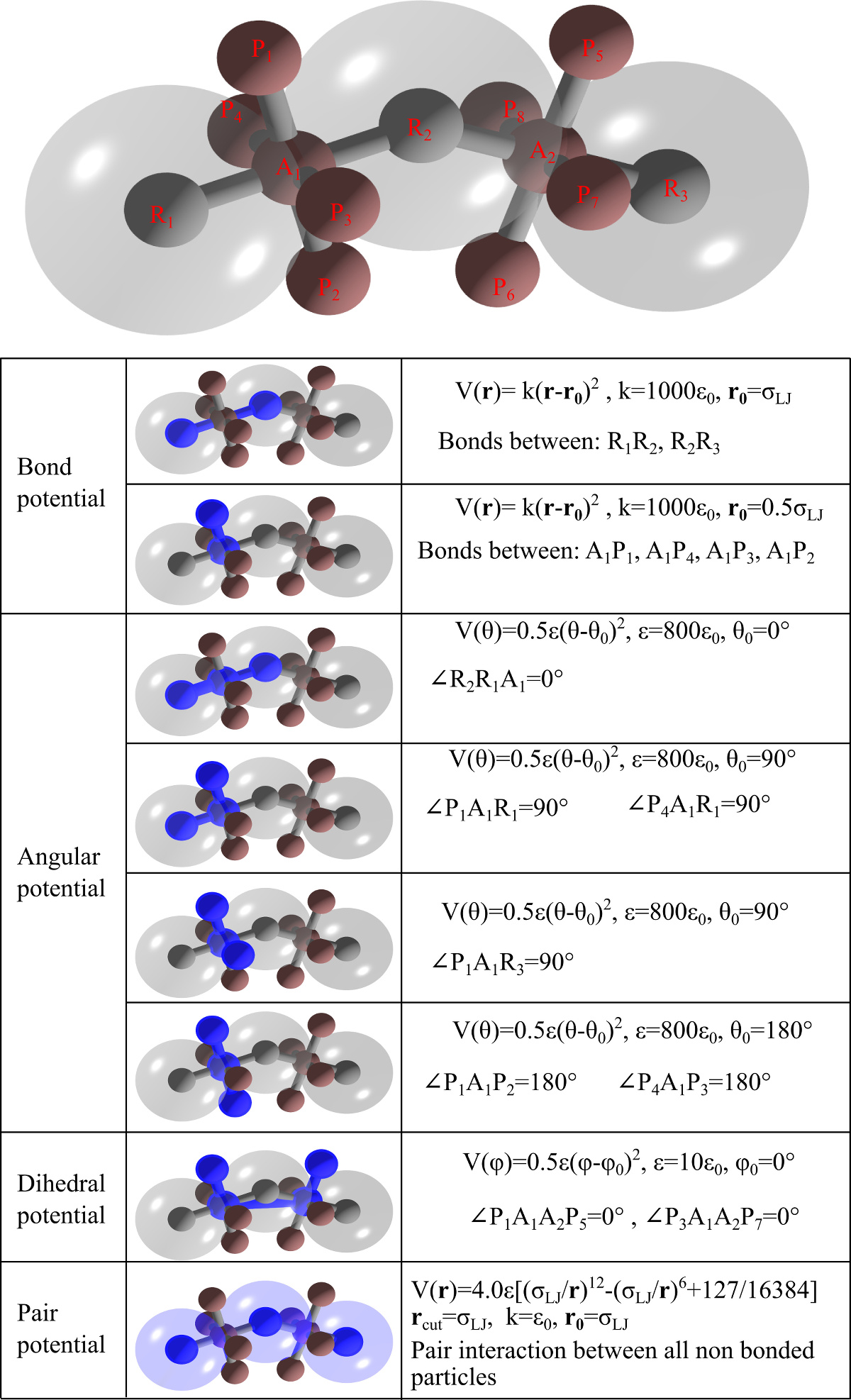


Figure SI1: Potentials involved in the construction of the worm-like chain model with bending and torsional resistance.

The chain is composed of real beads (R), with large zones of excluded volume shown as semitransparent spheres, and auxiliary beads that are needed to introduce torsional resistance via dihedral potentials. There are two types of auxiliary beads: axial (A) and periaxial (P). The auxiliary beads do not have any excluded volume interactions. The HOOMD software is unstable when some of the beads have too small mass, for this reason we set the mass of auxiliary beads to 1/10 of the mass of real beads. However, the mass of auxiliary beads only affects the dynamics of modelled molecules but not their equilibrium shapes. All bonding, angular and excluded volume potentials acting on modelled molecules are listed and specified together with drawings highlighting (in blue) the sets of beads involved in defining a given potential. In the figure we indicate all the relevant potentials for the basic element of our chain but the same applies to all consecutive portions of modelled chains.


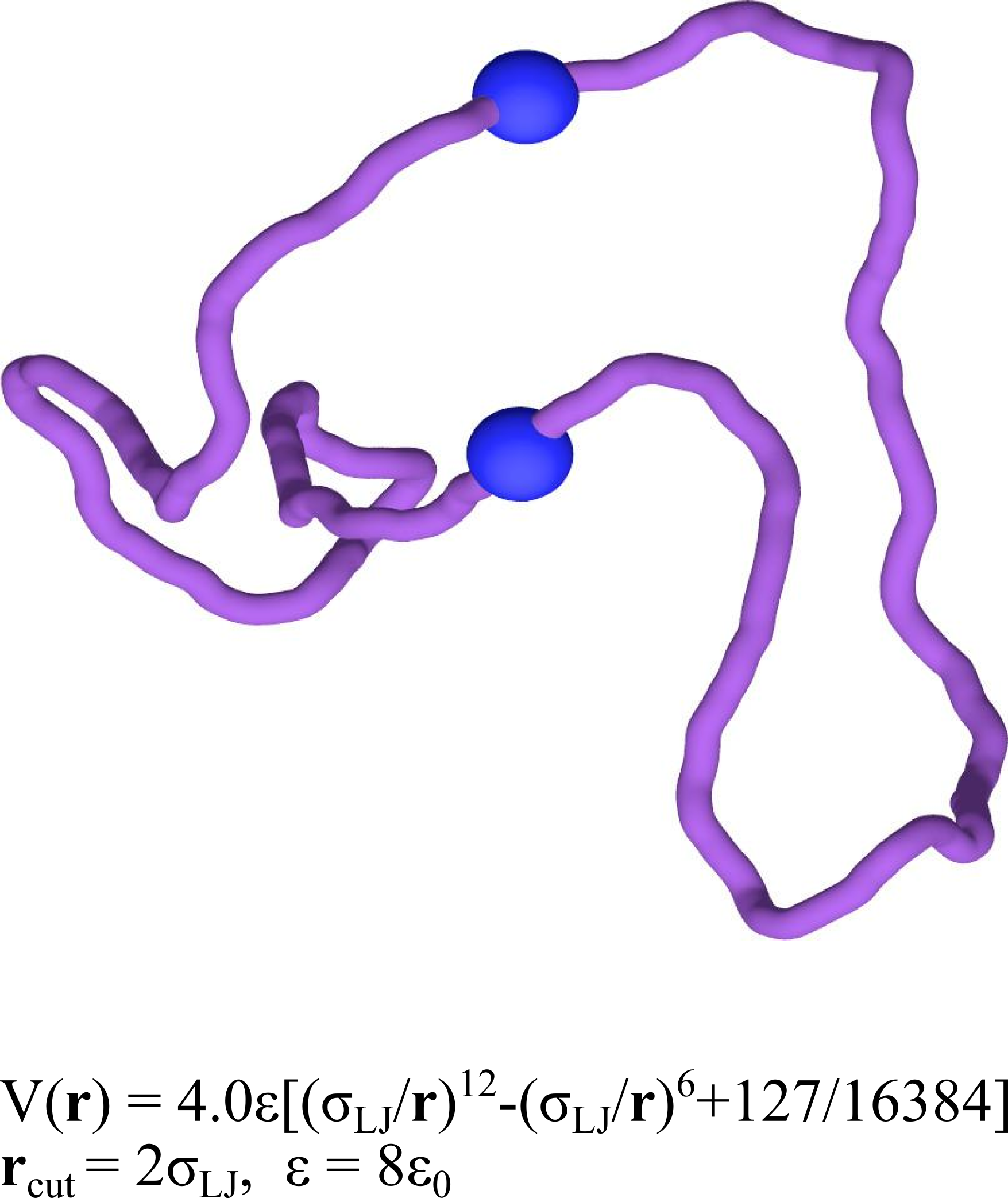


Figure SI2: Lennard-Jones potential used to model enhancer-promoter interactions.

For simulations testing the effect of crowding on enhancer-promoter interaction (presented in Fig.7), in addition to all potentials specified in Fig. S1, we introduced Lennard-Jones attractive potential acting on two beads placed 180° apart on the circular map of modeled DNA ring. To reflect short-range protein-protein interactions the potential was cut at the distance corresponding to 6 nm i.e. beyond this distance the two beads representing enhancer and promoter were not experiencing any attraction nor repulsion.


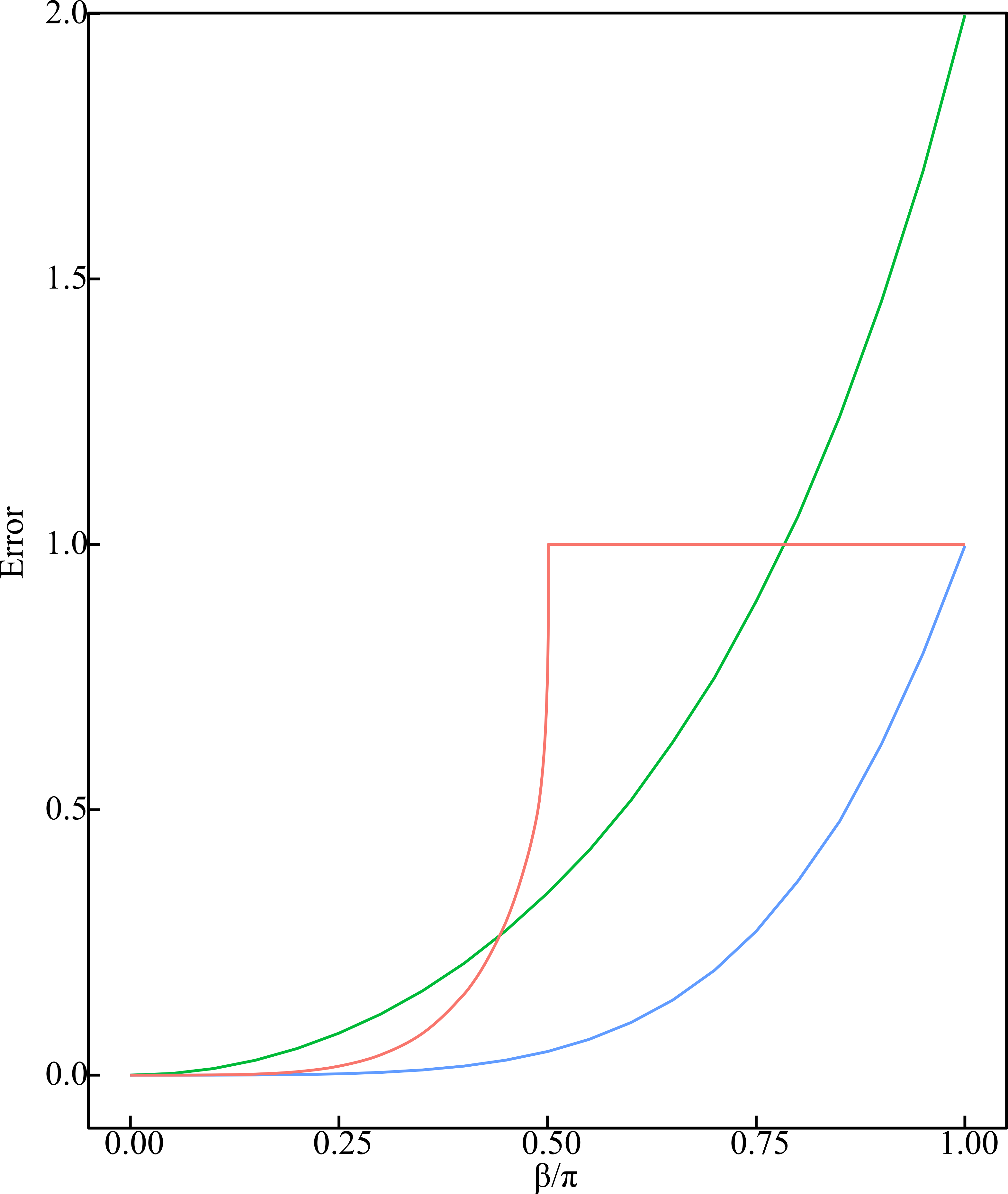


Figure SI3: Graph of the maximal possible error in the estimation of the twist angle based on measurements of dihedral angles in models proposed earlier by Benedetti et al., (30,48) (blue line), Brackley et al., (31) (red line) and the current model (see Fig. S1) (blue line). The higher is the bending angle (β/π), the less precise is the approximation of the actual twist angle by the dihedral angles that can be defined for beaded chain models using such software as HOOMD or LAMMPS. In addition, the error depends on the actual position of dihedral angles with respect of the plane defined by the local bend. The graphs are for least favorable positions of dihedral angles. Our current model is more accurate than the previous ones as it combines two advantages of earlier models. It uses two dihedral angles (as proposed by Brackley et al., 31) and places the reference frames used to determine dihedral angles between two consecutive chain beads (as proposed by Benedetti et al, (30,48). That placement decreases the deflection angle between the two frames and makes it that even for the bending angle of 90° the dihedral angle is always defined.
